# Cone-Beam Computed Tomography as a Diagnostic Method for Determination of Gingival Thickness and Distance between Gingival Margin and Bone Crest

**DOI:** 10.1155/2015/142108

**Published:** 2015-03-31

**Authors:** Germana Jayme Borges, Luis Fernando Naldi Ruiz, Ana Helena Gonçalves de Alencar, Olavo César Lyra Porto, Carlos Estrela

**Affiliations:** ^1^Faculty of Dentistry, UniEvangélica University Center, 75083-515 Anápolis, GO, Brazil; ^2^Faculty of Dentistry, Goiás Federal University, 74690-900 Goiânia, GO, Brazil

## Abstract

The objective of the present study was to assess cone-beam computed tomography (CBCT) as a diagnostic method for determination of gingival thickness (GT) and distance between gingival margin and vestibular (GMBC-V) and interproximal bone crests (GMBC-I). GT and GMBC-V were measured in 348 teeth and GMBC-I was measured in 377 tooth regions of 29 patients with gummy smile. GT was assessed using transgingival probing (TP), ultrasound (US), and CBCT, whereas GMBC-V and GMBC-I were assessed by transsurgical clinical evaluation (TCE) and CBCT. Statistical analyses used independent *t*-test, Pearson's correlation coefficient, and simple linear regression. Difference was observed for GT: between TP, CBCT, and US considering all teeth; between TP and CBCT and between TP and US in incisors and canines; between TP and US in premolars and first molars. TP presented the highest means for GT. Positive correlation and linear regression were observed between TP and CBCT, TP and US, and CBCT and US. Difference was observed for GMBC-V and GMBC-I using TCE and CBCT, considering all teeth. Correlation and linear regression results were significant for GMBC-V and GMBC-I in incisors, canines, and premolars. CBCT is an effective diagnostic method to visualize and measure GT, GMBC-V, and GMBC-I.

## 1. Introduction

Deeper knowledge of the biological structure and morphological quality of healthy periodontal tissue helps to establish the diagnosis and prognosis of periodontal diseases [1]. Esthetic periodontal procedures prior to dental rehabilitation have become common. Periodontal plastic surgeries have been recommended aiming to improve gingival contours, increase the amount of keratinized tissue and improve its quality, cover areas of exposed tooth root, and correct gummy smile [2–4].

The biologic width is essential for the maintenance of gingival health [5, 6] and any violations of this space may induce the destruction of periodontal supporting tissues [7]. It is widely accepted that the standard distance between the gingival margin and the alveolar bone crest is 3.0 mm [8, 9], which has been adopted in prosthetic and surgical procedures, as well as in the maintenance of periodontally treated patients. However, the dimensions of the dentogingival junction have been broadly discussed in the literature [10–12]. The biologic width of molars, measured in cadavers, is significantly greater than that of anterior teeth [11]. In their clinical observations, Perez et al. [12] verified that the average distance between the free gingival margin and the alveolar bone crest was 3.7 mm, considering the facial surface of maxillary central incisors. Nonetheless, when measured in the buccal surface, this distance ranged from 3.0 to 5.0 mm.

The variations observed in the dimensions of the dentogingival complex frequently hamper a professional clinical evaluation. The diagnostic methods normally used include periapical and interproximal X-rays, as well as bone probing [13–15]. Due to the limitations and inaccuracy of these exams [16] the overall treatment may be jeopardized [17].

The measurements of dentogingival junction using bone probing have been proved to be similar to histometric measurements [18, 19]. Nevertheless, anatomic crown length, soft and hard tissue thickness, and location of dentin-enamel junction are still controversial [20, 21].

Similarly, a deeper knowledge of the morphology of gingival tissue is of paramount importance for planning, execution, and prognosis of periodontal treatment. Ochsenbein and Ross [22] classified the gingival tissue into two main types, one scalloped and thin and the other flat and thick. However, some patients present characteristics of both tissue phenotypes, which suggests an intermediary periodontal biotype [23, 24].

Severity of signals and symptoms of periodontal disease may be related to the type of periodontium. In patients presenting with thick periodontium, the inflammation caused by bacterial plaque can cause periodontal pockets, whereas patients with the thin type can have gingival recessions [25]. Therefore, besides influencing the results of basic periodontal treatment [26], periodontal biotype interferes in root coverage procedures [27] and dental implant esthetics [28].

Several methods have been used to measure the thickness of gingival tissue [29], among which are the direct method or transgingival probing (TP) [30, 31], the method using ultrasound (US) [25, 32, 33], and, more recently, cone-beam computed tomography (CBCT) [34, 35].

TP presents limitations due to the low precision of periodontal probes with millimeter indentations and because it is an invasive procedure, which provokes discomfort for patients, therefore requiring local anesthesia [31].

Although US [25, 32, 33, 36] seems to be an effective method to measure gingival thickness (GT) [37], it is difficult to determine a correct and reproducible position to calibrate the equipment. Another disadvantage lies in the fact that this device does not allow a panoramic view of gingival/periodontal structures or the analysis of their relationships.

CBCT is a CT technology with emission of conic X-ray beams presenting limited emission of radiation. Its clinical applications permitted numerous discussions and advances in planning and diagnosing alterations in the maxillofacial region [34, 35, 38–42]. Nonetheless, one of its limitations is related to the difficulty of establishing limits between soft tissues and the vestibular bone crest. In order to lessen this disadvantage, Januário et al. [34] suggested that the patients should use a labial retractor during the exam. The usage of the labial retractor favoured the visualisation and measurement of soft and hard structures of the periodontium and allowed the clinician to assess the relationship between the periodontal structures.

Given that the knowledge of dentogingival complex dimensions and thickness of gingival tissue is a major aspect of the periodontal treatment, the objective of the present study was to assess CBCT as a diagnostic method of GT and the distance between the gingival margin to vestibular (GMBC-V) bone crest and gingival margin to interproximal bone crest (GMBC-I) comparing clinical measurements with those using CBCT.

## 2. Materials and Methods

### 2.1. Sample Selection

This study was approved by the Research Ethics Committee of the Universidade Federal de Goiás (protocol number 272/2011) and all the participants signed a free informed consent form. A group of 29 patients with complaints of gummy smile and indication of esthetic crown lengthening was selected for the study at the Clinic of the Dentistry School at the Universidade Federal de Goiás.

In addition to gummy smile, the inclusion criteria were as follows: no smoking, no drug abuse, no systemic complications or allergy history, nonpregnant women, over 18 years old, and presence of all maxillary teeth, except for third molars. The exclusion criteria were as follows: previous periodontal surgical procedures, use of medicines that change periodontal tissues, such as cyclosporine A, calcium channel blockers, phenytoin, and codes 3 and/or 4 score of the Periodontal Screening and Recording (PSR) system.

### 2.2. Initial Preparation of Patients

After anamnesis and PSR, all the patients received individualised oral hygiene instructions. Those scoring a PSR code 2 underwent scaling and root planing. Dental photography for case documentation and silicon impressions (Zhermack, Zetaplus, Badia Polesine, Italy) of maxillary arch to obtain a study model were also performed in order to build a tomographic and a clinical guides aiming to standardise GMBC-V, GMBC-I, and GT measured by different methods.

### 2.3. Laboratory Procedures: Tomographic and Clinical Guides

**Figure 1:**
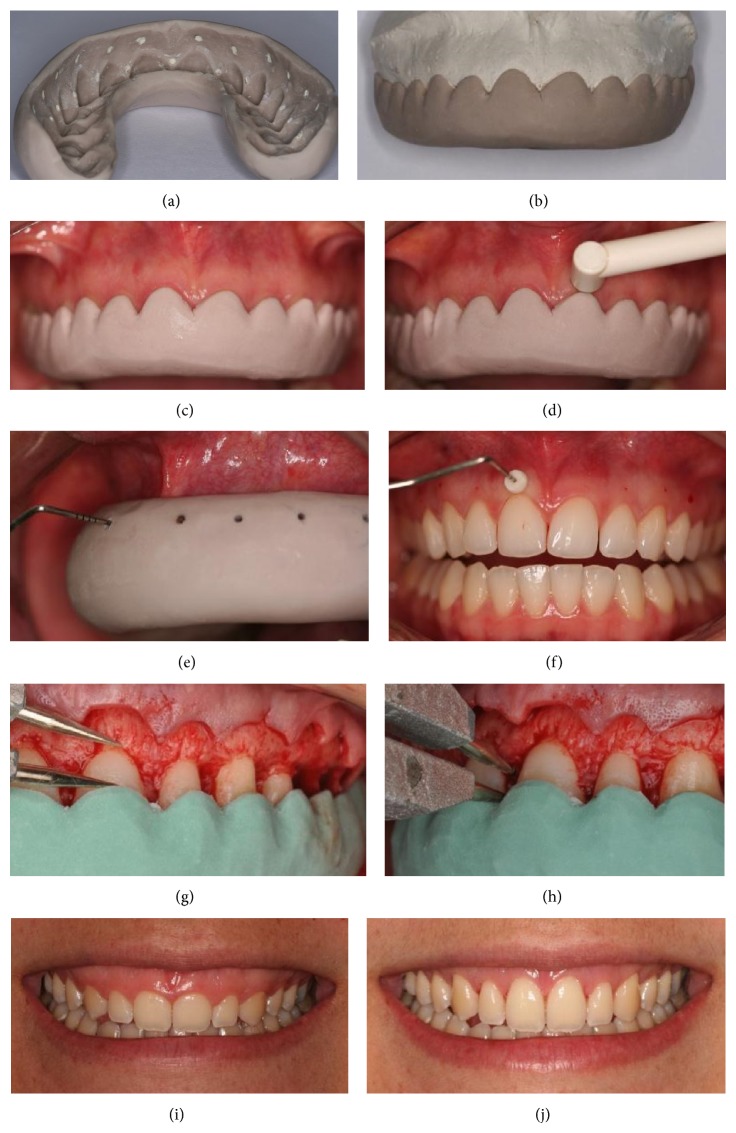
(a) Tomographic guide showing the marks made at the tip of each interproximal papilla and 3.0 mm above the gingival margin of each tooth. (b) Clinical guide following the contours of the gingival margin and the tips of the interproximal papillae. (c) Positioning the clinical guide to measure gingival thickness (GT) using ultrasound (US). (d) Positioning the US to measure GT. (e) Marking the point to measure GT. (f) Silicon limiter in contact with the external surface of the gum to measure GT. (g) Measuring the distance between the gingival margin and the vestibular bone crest (GMBC-V) at the highest point from the clinical guide margin to the vestibular bone crest. (h) Measuring the distance between the gingival margin and the interproximal bone crest (GMBC-I) from the point in the clinical guide which represents the tip of the interproximal papilla to the interproximal bone crest. (i) Patient's gummy smile. (j) Patient's smile after surgery.

The internal face of the silicon impression used as the tomographic guide was marked both at the tip of each interproximal papilla and 3.0 mm above the gingival margin of each tooth, using a 1.0 mm diameter diamond round bur. These marks were filled with radiopaque material (zinc oxide eugenol cement) and used as reference to measure GMBC-V, GMBC-I, and GT in CBCT imaging (Figure 1(a)).

The silicon impression used as the clinical guide was cut, using a scalpel blade number 15C, following the contours of the gingival margin and the tips of the interproximal papillae to help measure GMBC-I during surgery to lengthen the clinical crowns (Figure 1(b)).

### 2.4. Complementary Exam: CBCT

After the initial preparation, all patients underwent CBCT in a private clinic (Centro Integrado de Radiologia Odontológica (CIRO), Goiânia, GO, Brazil). During this exam, the patients used a labial retractor [34] and the tomographic guide. CBCT images were acquired using the i-CAT Cone-Beam 3D Imaging System (Imaging Sciences International, Hatfield, PA, USA), at 120 KVp and 3.8 mA for 40 s (voxel size: 0.25 mm; grayscale: 14 bits; focal spot: 0.5 mm; field of view: 6.0 cm) and a single 360° image rotation. The images were processed by Xoran CAT software, version 3.1.62 (Xoran Technologies, Inc., Ann Arbor, MI, USA), in a computer with Microsoft Windows XP Professional SP-2 program (Microsoft Corporation, Redmond, WA, USA), with an Intel Core 2 Duo 1.86 Ghz-6300 processor (Intel Corporation, San Jose, CA, USA), a video card NVIDIA GeForce 6200 with TurboCache (NVIDIA Corporation, Santa Clara, CA, USA) and LCD monitor EIZO FlexScan S2000, resolution 1600 × 1200 pixels (Eizo Nanao Corporation, Ishikawa, Japan). After reconstruction of raw data, the digital imaging and communications in medicine (DICOM) files were generated for each patient.

### 2.5. Clinical Procedures

#### 2.5.1. GT Measurement Using US

The clinical guide was placed in the patient's mouth and the transducer of the US (Krupp SDM, Austenal Medizintechnik, Cologne, Germany) was positioned above the edge of the guide on the vestibular gingival tissue of each tooth (Figures 1(c) and 1(d)). GT of maxillary incisors (I), canines (C), premolars (PM), and first molars (FM) was measured. The measurements were taken three times, by a single periodontist with over 5 years of experience, and registered.

#### 2.5.2. GT Measurement Using TP

This exam was carried out during surgery to lengthen the clinical crowns prior to raising the flap. After local anesthesia, the tomographic guide was positioned in the patient's mouth and a periodontal probe was used to penetrate it and mark the soft tissue on the vestibular surface of each tooth (Figure 1(e)). After that, a periodontal probe with a silicon limiter was positioned perpendicularly to the long axis of the tooth, at the point previously marked, penetrating the gingival tissue until meeting resistance of vestibular bone plate or dental structure (Figure 1(f)). The silicon limiter was adjusted to be in direct contact with the external surface of the gum. Finally, the probe was carefully removed and the penetration length was verified with a digital caliper reading to 0.01 mm (Mitutoyo MTI Corporation, Tokyo, Japan).

#### 2.5.3. Transsurgical Clinical Evaluation (TCE) of GMBC-V and GMBC-I

After measuring GT, a gingival collar was removed between tooth 16 and tooth 26. Using a syndesmotome, a total thickness flap was carefully elevated at the vestibular side exposing the bone crest. At this moment of the procedure, the clinical guide was positioned and GMBC-V was verified from the highest point of the gingival margin to the vestibular bone crest using an aluminum blunt tip compass (Jon Comércio de Produtos Odontológicos, São Paulo, SP, Brazil) placed parallel to the long axis of each tooth (Figure 1(g)). Using a digital caliper, GMBC-V was measured and registered. Likewise, GMBC-I was verified from the tip of the papilla to the interproximal bone crest using an aluminum blunt tip compass, measured using the digital caliper, and registered. The measurements were taken three times, by a single periodontist with over 5 years of experience (Figure 1(h)). After all measurements, osteoplasty and osteotomy were performed to lengthen the clinical crowns; the flap was repositioned and sutured.

#### 2.5.4. Analysis of CBCT Imaging

GT, GMBC-V, and GMBC-I measurements were taken in 1.0 mm thick transversal vestibular-palatal slices with 1.0 mm spacing between contiguous slices. The measurement tool supplied by the scanner manufacturer (Xoran CAT software version 3.1.62) and filters to refine and enhance the image were used to ensure the precision of each measurement. All these measurements were taken by a single radiologist with over 5 years of experience in interpreting CBCT imaging.

#### 2.5.5. GT Measurement Using CBCT Imaging

**Figure 2:**
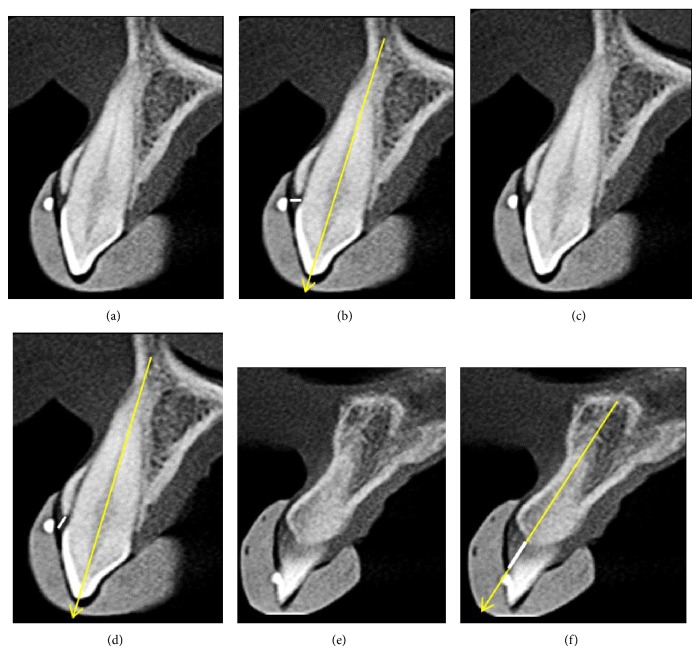
(a) Transversal slice of tooth 13 evidencing hyperdense and round mark. (b) Measuring gingival thickness (GT, white line, perpendicular to the long axis of the tooth). (c) Transversal slice of tooth 13 evidencing hyperdense and round mark. (d) Measuring the distance between the gingival margin and the vestibular bone crest (GMBC-V, white line, parallel to the long axis of the tooth, traced from the gingival margin to the vestibular bone crest). (e) Transversal slice of the region between teeth 11 and 12 with hyperdense and round mark at the tip of the interproximal papilla. (f) Measuring the distance between the gingival margin and the interproximal bone crest (GMBC-I, white line).

The reference for this measurement was the mark in the tomographic guide made 3.0 mm above the gingival margin of each tooth (Figure 2(a)). The transversal vestibular-palatal slice in which this mark was evident in its largest dimension was used for this measurement. In the center of the mark, a line was traced perpendicularly to the long axis of the tooth, similar to the insertion of the periodontal probe clinically performed, and the distance from the external side of the gingival tissue to the bone crest or dental structure was measured (Figure 2(b)).

#### 2.5.6. GMBC-V Measurement Using CBCT Imaging

In the same transversal vestibular-palatal slice used to measure GT, GMBC-V was measured (Figure 2(c)). Following the long axis of the tooth, a parallel line was traced from the gingival margin to the vestibular bone crest (Figure 2(d)).

#### 2.5.7. GMBC-I Measurement Using CBCT Imaging

The reference for this measurement was the mark in the tomographic guide made at the tip of each interproximal papilla. The transversal vestibular-palatal slice in which this mark was evident, hyperdense, and round was used for this measurement (Figure 2(e)). A line was traced in the center of the alveolar ridge and another line was traced parallel to it from the gingival margin to the interproximal bone crest (Figure 2(f)).

### 2.6. Statistical Analysis

Mean and standard deviation of GT, GMBC-V, and GMBC-I were calculated. The difference between the measurements performed with CBCT, TP, US, and TCE was calculated using the independent *t*-test or the Mann-Whitney test and ANOVA Tamhane's post hoc test. The relationship between the measurements performed with CBCT, TP, US, and TCE was assessed by the Pearson correlation coefficient and simple linear regression. The significance level was *P* < 0.05. The statistical analysis was carried out using the Statistical Package for the Social Sciences software, version 20 (SPSS, Chicago, IL, USA).

## 3. Results

For the present study, 29 patients were selected, 27 females and 2 males, with mean age of 27 years (18 to 49 years). GT and GMBC-V were measured in a total of 348 maxillary teeth, 116 I, 58 C, 116 PM, and 58 FM. GMBC-I was measured in 377 regions, 29 between central incisors (CI-CI), 58 between central and lateral incisor (CI-LI), 58 between lateral incisor and canine (LI-C), 58 between canine and premolar (C-PM), 58 between premolars (PM-PM), 58 between premolar and first molar (PM-FM), and 58 between first molar and second molar (FM-SM).

### 3.1. GT Measurement

Table 1 shows the mean and standard deviation of GT measurements obtained by CBCT imaging, TP, and US, considering teeth groups (I, C, PM, and FM) and all the teeth. Significant statistical differences were observed among the three methods considering teeth groups and all the teeth. Also, significant statistical difference was found in the individual groups of I and C between the measurements obtained by CBCT and TP and obtained by TP and US, but no difference was observed between CBCT and US. In the individual groups of PM and FM, no significant statistical difference was found between the measurements obtained by CBCT and TP, while significant statistical differences were observed between TP and US as well as between CBCT and US. The highest means of GT measurements were obtained by TP compared to the other methods.

A significant positive correlation was observed between TP and CBCT (*P* < 0.05) for GT measurements obtained for all the teeth (*r* = 0.401) and teeth groups (I, *r* = 0.371; C, *r* = 0.442; PM, *r* = 0.466; M, *r* = 0.300). The same was observed between CBCT and US (all the teeth, *r* = 0.475; I, *r* = 0.416; C, *r* = 0.532; PM, *r* = 0.549; FM, *r* = 0.533) and between TP and US (all the teeth, *r* = 0.430; I, *r* = 0.440; C, *r* = 0.517; PM, *r* = 0.442; M, *r* = 0.295). The linear regression analysis showed significance for all teeth groups and all the teeth between TP and CBCT, between CBCT and US, and between TP and US (*P* < 0.05).

### 3.2. GMBC-V Measurement

Using TCE as the standard reference method, significant statistical differences were observed between TCE and CBCT for GMBC-V measurements obtained for all the teeth and teeth groups. CBCT registered higher values for GMBC-V measurements than TCE. In both methods, GMBC-V measurements were higher for I, C, PM, and FM (Table 2).

A significant positive correlation was observed between TCE and CBCT (*P* < 0.05) for measurements obtained for all the teeth (*r* = 0.692) and I (*r* = 0.442), C (*r* = 0.564), and PM (*r* = 0.552), whereas the M group did not present a significant correlation (*r* = 0.014; *P* = 0.915). The linear regression analysis between TCE and CBCT showed significance for I, C, PM, and all the teeth (*P* < 0.05), but not for the FM group (*P* = 0.915).

### 3.3. GMBC-I Measurement

Using TCE as the standard reference method, significant statistical differences were observed between TCE and CBCT for GMBC-I measurements obtained for CI-CI, CI-LI, LI-C, C-PM, PM-PM, PM-FM, and FM-SM regions, also considering all the interproximal regions. In all the analyses, CBCT registered higher values for GMBC-I measurements than TCE. In both methods, the highest mean GMBC-I measurements occurred in the C-PM region, 3.68 mm and 3.16 mm, respectively (Table 2).

A significant positive correlation was observed between TCE and CBCT (*P* < 0.05) for measurements considering all the interproximal regions (*r* = 0.398) and the CI-CI (*r* = 0.393), LI-LI (*r* = 0.363), LI-C (*r* = 0.278), C-PM (*r* = 0.473), PM-PM (*r* = 0.448), and PM-FM regions (*r* = 0.378), but not for the FM-SM region (*r* = 0.239; *P* = 0.071). The linear regression analysis between TCE and CBCT did not show significance only for the M-M region (*P* = 0.071).

## 4. Discussion

The predictability of periodontal therapy results is better estimated when it is based on deeper knowledge of the dimensions of dentogingival structures and annexes. Aiming to assess these measurements and shapes, several researches have been conducted to monitor and quantify gingival and periodontal alterations, which implies the use of precise methods. An expressive improvement has been achieved with CBCT, a new reliable resource for diagnostic and therapeutic treatment plan purposes, which allows viewing three-dimensional images [21, 38].

In the present study, significant differences were registered between TP and CBCT for incisives and canines, with lower means found for the latter. In contrast, evaluating gingival thickness in cryopreserved and thawed teeth of human cadavers, measured at 2.0 mm below the alveolar bone crest, using caliper and CBCT, Fu et al. [20] did not observe differences between incisives and canines. The differences between the studies might be explained by the difficulty of establishing limits between soft tissues and the vestibular bone crest in CBCT imaging. Furthermore, the measurements were carried out in vivo in our study and ex vivo in the other experiment.

The posterior localization of premolars and molars contributes to the difficulty of their clinical assessment. However, the results of this study showed that clinical gingival thickness measurements were similar to those obtained using CBCT, which suggests an advantage of the latter to evaluate this region.

The knowledge of gingival thickness dimensions favours the planning of periodontal and restorative procedures, which may influence the prognosis. In this study, the mean gingival thickness obtained using CBCT was 1.17 ± 0.26 mm for incisives and 1.08 ± 0.28 mm for canines. Similar results were registered by Batista et al. [41] and La Rocca et al. [43] For molars, the mean gingival thickness found in our study was 1.32 ± 0.54 mm, higher than that reported by Ueno et al. [44] (1.13 ± 0.88 mm), who measured gingival thickness in human cadavers using MSCT.

The results found for maxillary incisives and canines using TP, in the present study, were 1.34 ± 0.31 and 1.22 ± 0.27 mm, respectively. Savitha and Vandana [37] reported a mean of 1.08 ± 0.42 mm for maxillary and mandibular incisives and canines. In our study, significant differences were observed between gingival thickness measurements using TP and US, similar to the findings of Savitha and Vandana [37]. Although US is a noninvasive diagnostic method and relatively easy to use, positioning the transducer is difficult, mainly in the posterior region, and this may interfere in the reproducibility of these measurements [45].

The distance between the gingival margin and the alveolar bone crest, which encompasses the measurements of gingival sulcus, junctional epithelium, and conjunctive attachment should be taken into consideration in restorative and surgical procedures. In the present study, significant difference was observed for the distance between the gingival margin and the vestibular and interproximal bone crests measured by TCE and CBCT, considering all the teeth and the groups of teeth analysed, and the means were higher using the latter. Also, the mean distance between the gingival margin and the vestibular bone crest considering all the teeth was 2.54 ± 0.85 mm, similar to the results reported by Gargiulo et al. [10] in a histological study in cadavers. The mean dimensions of the dentogingival structures described by the authors, named physiological dentogingival unit, were 0.97 mm for the junctional epithelium, 1.07 mm for the conjunctive attachment, and 0.69 mm for the gingival sulcus, whereas the total length of the dentogingival complex was 2.73 mm. Xie et al. [46] reported a lower histological measurement (2.17 ± 0.18 mm), but they considered the biological distance, that is, the combined measurement of the conjunctive attachment and the junctional epithelium.

Considering the teeth groups, the means found for both the distance between the gingival margin and the vestibular and interproximal bone crests in the present study using TCE were inferior to those registered by Perez et al. [12] using TP, a fact that might be explained by the differences between the methods employed.

In the present study, a tomographic guide and a clinical guide were developed aiming to standardise the position to calibrate the equipment both clinically and in the images. However, differences between the measurements obtained in CBCT images of TCFC and those obtained clinically. These results may be justified by the interferences related to technical matters regarding the generation of the images.

Voxel size seems to be critical in the evaluation of bone height around the teeth [47]. Areas with thin vestibular bone plates are susceptible to discrepancies [48], since they are difficult to be visualised and measured, even using a labial retractor, as in the current study. The position of the tooth in the arch may also influence the precision of the bone image [49], and, in this regard, the molar region is more complex to be assessed.

Despite the differences observed, the measurements obtained using different methods in this study were correlated and presented significant positive correlation and linear regression for I, C, and PM groups. This demonstrates that when the variable increases clinically, the same remains true for CBCT images. Therefore, CBCT can contribute to the diagnosis and planning of periodontal and restorative procedures.

Esthetic crown lengthening requires careful planning to determine the best technique and the correct amount of soft and hard tissues to be removed, thus avoiding deficient or excessive removal, in order to ensure the stability of the results achieved in the immediate postoperative period [50]. Normally, the treatment plan for gummy smile is based on clinical and imaging assessments [17, 51] to determine the following aspects: gingival tissue thickness and height, bone thickness, distance between the gingival margin and the vestibular and interproximal bone crests, and distance between the bone crest and the dentin-enamel junction. Nonetheless, both in clinical and imaging assessments, certain limitations have been identified for the acquisition of these measurements. CBCT imaging contribution in this field lies in the possibility of visualising and measuring these structures and the interrelationships of the tissues in three dimensions, which may minimize possible diagnostic and treatment planning errors.

The clinical value of dynamic image analysis brought new perspectives of noninvasive diagnostic methods, which enrich the establishment of diagnosis, planning, and successful results based on rigorous comparisons. The precision of the results regarding visual features of soft and hard tissues, the exposure to radiation, and the cost benefit of CBCT imaging are still challenging. Further studies are necessary to minimize these variables in order to ensure the promising results of three-dimensional images.

## 5. Conclusion

CBCT is an effective diagnostic method to visualize and measure GT, GMBC-V, and GMBC-I, presenting measurements correlated to those obtained clinically and, therefore, contributing to a better planning of esthetic procedures in periodontics.

## Figures and Tables

**Table 1:** Mean and standard deviation (sd) of gingival thickness (GT) measurements obtained by cone-beam computed tomography (CBCT) imaging, transgingival probing (TP), and the method using ultrasound (US).

Teeth group	Gingival thickness^1^ (mm)						
	CBCT		TP		US		*P*
	Mean ± sd	*n*	Mean ± sd	*n*	Mean ± sd	*n*	
Incisors	1.17 ± 0.26^a^	116	1.34 ± 0.31^b^	116	1.24 ± 0.32^a^	58	<0.05
Canines	1.08 ± 0.28^a^	58	1.22 ± 0.27^b^	58	1.03 ± 0.38^a^	58	<0.05
Premolars	1.19 ± 0.48^a^	116	1.23 ± 0.37^a^	116	1.01 ± 0.43^b^	116	<0.05
Molars	1.32 ± 0.54^a^	58	1.39 ± 0.42^a^	58	1.06 ± 0.36^b^	58	<0.05
All the teeth	1.18 ± 0.40^a^	348	1.29 ± 0.35^b^	348	1.10 ± 0.39^c^	348	<0.05

^1^Different letters in the lines indicate significant statistical differences (*P* < 0.05) by Tamhane's test.

**Table 2:** Mean and standard deviation (sd) of distance between the gingival margin and vestibular bone crests (GMBC-V) gingival margin and interproximal bone crests (GMBC-I) obtained by transsurgical clinical evaluation (TCE) and cone-beam computed tomography.

Teeth group	Distance between gingival margin and vestibular bone crest (mm)				
	TCE		CBCT		*P*
	Mean ± sd	*n*	Mean ± sd	*n*	
Incisors	3.14 ± 0.72	116	3.55 ± 0.61	116	<0.05
Canines	2.90 ± 0.91	58	3.25 ± 0.60	58	<0.05
Premolars	2.08 ± 0.57	116	2.42 ± 0.44	116	<0.05
Molars	1.93 ± 0.45	58	2.35 ± 0.43	58	<0.05
All the teeth	2.54 ± 0.85	348	2.93 ± 0.75	348	<0.05

Teeth region	Distance between gingival margin and interproximal bone crest (mm)				
	TCE		CBCT		*P*
	Mean ± sd	*n*	Mean ± sd	*n*	

CI-CI (11–21)	3.11 ± 0.74	29	3.51 ± 0.53	29	<0.05
CI-LI (12-11; 22-21)	2.90 ± 0.74	58	3.32 ± 0.45	58	<0.05
LI-C (13-12; 23-22)	3.05 ± 0.61	58	3.48 ± 0.50	58	<0.05
C-PM (14-13; 24-23)	3.16 ± 0.65	58	3.68 ± .57	58	<0.05
PM-PM (15-14; 25-24)	2.90 ± 0.66	58	3.20 ± 0.52	58	<0.05
PM-M (16-15; 26-25)	2.83 ± 0.51	58	3.02 ± 0.42	58	<0.05
M-M (17-16; 27-26)	2.89 ± 0.65	58	3.30 ± 0.48	58	<0.05
All the regions	2.97 ± 0.65	377	3.34 ± 0.53	377	<0.05

Independent samples *t*-test.
